# Microfluidic Biochips for Single‐Cell Isolation and Single‐Cell Analysis of Multiomics and Exosomes

**DOI:** 10.1002/advs.202401263

**Published:** 2024-05-20

**Authors:** Chao Wang, Jiaoyan Qiu, Mengqi Liu, Yihe Wang, Yang Yu, Hong Liu, Yu Zhang, Lin Han

**Affiliations:** ^1^ Institute of Marine Science and Technology Shandong University Qingdao 266237 China; ^2^ Department of Periodontology School and Hospital of Stomatology Cheeloo College of Medicine Shandong University Jinan 250100 China; ^3^ State Key Laboratory of Crystal Materials Shandong University Jinan 250100 China; ^4^ Shandong Engineering Research Center of Biomarker and Artificial Intelligence Application Jinan 250100 China

**Keywords:** isolation quality indicators, microfluidic chips, multiomics and exosome applications, single cells

## Abstract

Single‐cell multiomic and exosome analyses are potent tools in various fields, such as cancer research, immunology, neuroscience, microbiology, and drug development. They facilitate the in‐depth exploration of biological systems, providing insights into disease mechanisms and aiding in treatment. Single‐cell isolation, which is crucial for single‐cell analysis, ensures reliable cell isolation and quality control for further downstream analyses. Microfluidic chips are small lightweight systems that facilitate efficient and high‐throughput single‐cell isolation and real‐time single‐cell analysis on‐ or off‐chip. Therefore, most current single‐cell isolation and analysis technologies are based on the single‐cell microfluidic technology. This review offers comprehensive guidance to researchers across different fields on the selection of appropriate microfluidic chip technologies for single‐cell isolation and analysis. This review describes the design principles, separation mechanisms, chip characteristics, and cellular effects of various microfluidic chips available for single‐cell isolation. Moreover, this review highlights the implications of using this technology for subsequent analyses, including single‐cell multiomic and exosome analyses. Finally, the current challenges and future prospects of microfluidic chip technology are outlined for multiplex single‐cell isolation and multiomic and exosome analyses.

## Graphical abstract

This review offers detailed guidance on microfluidic chip technologies for single‐cell isolation and analysis. It details the isolation mechanisms, features, and cellular effects of various microfluidic chips. Additionally, the review highlights the critical contributions of these technologies to advancing single‐cell multiomics and exosome analysis across diverse scientific disciplines. Lastly, it discusses present challenges and future prospects for enhancing these technologies.

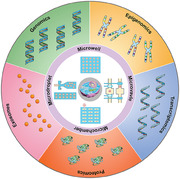

## 1 Introduction

Cells contain various biological components, such as DNA, RNA, proteins, and exosomes, which perform complex intracellular and intercellular functions and jointly maintain the normal physiological activities of cells.^[^ ^1^, ^2^, ^3^, ^4^, ^5^ ^]^ There are complex connections among them.^[^ ^6^, ^7^ ^]^ However, their roles remain controversial and difficult to elucidate because the microenvironment can affect the expression of these messages, and it is challenging to determine the cells from which these biological messages originate, as even the same cells produce different biological messages in different local environments.^[^ ^8^, ^9^ ^]^ Therefore, analytical techniques are needed not only to detect this biological information but also to perform complete multiomics and exosome analysis from live cells to cell lysates with single‐cell resolution, which is important to fully understand the complexity of biological systems.

However, biological information is usually present at very low concentrations in single cells.^[^ ^10^ ^]^ Therefore, any detection method requires high sensitivity and specificity, preferably with multiplexing. In recent years, microfluidic chip technology has developed rapidly and has many advantages over traditional technologies.^[^ ^11^ ^]^ Typical microfluidic structures have dimensions of tens to hundreds of micrometers, which are comparable to the size of a single cell, and can handle small amounts of sample reagents (nL–pL),^[^ ^12^ ^]^ enabling highly sensitive analysis. A relatively closed space reduces interference from the external environment and indirectly improves the specificity. The emergence of microfluidic chips with various structures and strategies has enabled multiple quantitative detections.^[^ ^13^, ^14^ ^]^ Although there has been made in the quantitative analysis of target molecules based on microfluidic technology and single‐cell isolation, which is an important prerequisite for single‐cell analysis, it is still challenging to isolate high‐quality single cells with natural expression profiles.

Traditional single‐cell isolation techniques include limiting dilution,^[^ ^15^ ^]^ laser capture microdissection (LCM),^[^ ^16^ ^]^ micromanipulation,^[^ ^17^, ^18^ ^]^ and fluorescence‐activated cell sorting (FACS).^[^ ^19^ ^]^ Although these are well‐established techniques, they have both advantages and disadvantages. Owing to its simplicity, low cost, and good cell activity, limiting dilution has been widely used for decades to isolate monoclonal cells for antibody production.^[^ ^20^, ^21^ ^]^ However, owing to the separation principle of continuous equal division, obtaining single cells has statistical randomness and low efficiency. According to the Poisson distribution, the maximum efficiency of single‐cell separation was lower than 37%.^[^ ^15^ ^]^ Furthermore, microscopic imaging is necessary to determine the location of single cells, and the throughput is limited by the well plate.^[^ ^22^ ^]^ LCM, because of its ease of manipulation and in situ isolation, LCM is often used to isolate specific pure cells for subsequent analyses.^[^ ^16^ ^]^ However, manual selection and isolation processes severely limit throughput and samples often require fixation, which compromises cell integrity and activity. Studies that require cell viability (e.g., proteins and exosomes) cannot be performed. In particular, when contact‐based cell extraction methods are used, it cannot be determined whether adjacent cell debris is transferred to the matrix along with target cells.^[^ ^23^ ^]^ Micromanipulation is similar to LCM, and the targeted isolation of specific cells under a microscope is one of the main advantages of this technique. In addition, this method is applicable to various cell types and can selectively isolate live cells, including prokaryotic cells, from suspensions.^[^ ^24^ ^]^ with the advantages of flexible operation and good cell activity.^[^ ^17^, ^18^ ^]^ However, the manual process of obtaining single cells using this method limits single‐cell throughput, and additional observations of the target location are required to determine whether single cells are successfully transferred. Although the development of mechanical automation has made automated micromanipulation an alternative to manual work, its serial processing still limits single‐cell throughput.^[^ ^25^ ^]^ The advent of FACS has greatly increased single‐cell throughput. Such systems can generate up to 100000 droplets per second and analyze up to 70000 cells per second. They are often used to isolate heterogeneous cell samples and have increasing prospects for diagnosis and treatment.^[^ ^19^, ^26^, ^27^ ^]^ However, FACS also has certain limitations: the systems are typically bulky, mechanically complex, and expensive, and can only be used to analyze cells at fixed time points,^[^ ^28^ ^]^ cells must be in suspension, which leads to the loss of tissue structure and function in the adherent state.^[^ ^29^ ^]^ In addition, the sorting shear force and detection laser can cause damage to cell activity,^[^ ^30^ ^]^ the most important limitation is that the cells isolated by FACS must be at a certain concentration, and the total number of rare cells cannot be processed.^[^ ^19^ ^]^ The quality indicators, such as throughput, efficiency, space, and cell activity, of the above traditional single‐cell isolation technologies are compared and summarized in **Table** **1**. Over the past few years, numerous mature microfluidic chips have been developed for single cell isolation and analysis. Polydimethylsiloxane (PDMS) is the most widely used microfluidic material owing to its good light transmittance and low biological toxicity.^[^ ^31^ ^]^ However, no single‐cell chip can guarantee all excellent indicators simultaneously because some indicators are in conflict. When ensuring high‐activity single‐cell isolation, it is often necessary to increase throughput and efficiency as much as possible while ensuring the space and activity of cell isolation. However, owing to the existence of the Poisson distribution, the isolation efficiency is insufficient, and certain cells are lost. In contrast, improving isolation efficiency requires reducing the physical size, which further increases cell throughput but inevitably reduces the space and cell activity indicators.

**Table 1:** . Comparison of the isolation quality indicators of various traditional single‐cell isolation technologies.

Traditional technology	Throughput	Efficiency	Space	Cell activity
Limiting dilution	Medium	Low	High	High
Laser capture microdissection	Low	High	High	Low
Micromanipulation	Low	High	High	High
FACS	High	Medium	Low	Low

In the following sections, various single‐cell isolation and analysis techniques based on microfluidic technologies are discussed in detail. These include the working principles, separation mechanisms, chip characteristics, and their impact on cellular functional activities, particularly in the analysis of multiomics and exosomes. Relevant research progress and important results are introduced, and the advantages, disadvantages, and applicability of different chips in different research fields are summarized. Finally, we discuss the current challenges and future prospects of microfluidic chip technology in single‐cell analysis of multiomics and exosomes.

## 2 Single‐Cell Isolation Chip Technology

Single‐cell isolation chip technology can be classified according to the separation quality indicators, including throughput, efficiency, space, and cell activity. These quality indicators are important for subsequent single‐cell multiomics and exosome profiling applications because 1) throughput determines whether the technology is sufficient to provide the statistical analysis of big data bioinformatics required for modern applications; 2) efficiency determines whether rare cells can be processed; 3) space determines whether the cell culture medium needs to be continuously exchanged, whether the cells, especially adherent cells, have enough living space, and the throughput and sensitivity of detection; and 4) cell activity determines whether the correct single‐cell bioinformatics results are obtained.

In this section, existing chip technologies are classified according to their isolation quality indicators and discussed, with a focus on their working principles, separation mechanisms, structural characteristics, and ability to isolate single cells in terms of throughput, efficiency, activity, and space.

### 2.1 High‐Activity Culture and Multi‐Factor Detection Microchamber Single‐Cell Isolation Chip

**Figure 1:**
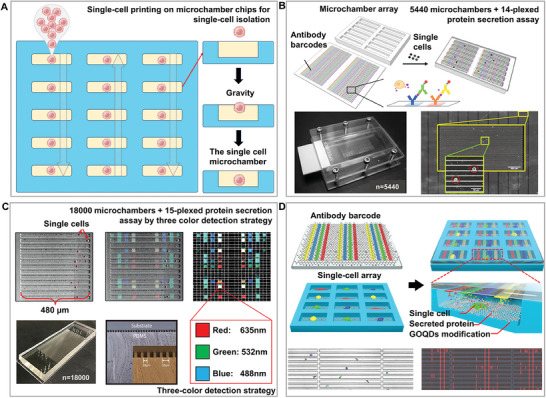
High‐activity micro‐barcode chamber chip. A). Single‐cell printing based on the microchamber chip technology: Gray arrows indicate the direction of single‐cell printing through the microchambers, highlighting the critical stages of single‐cell isolation and gravity deposition. B) High‐throughput microchamber isolation and multiplex protein analysis based on single‐cell printing. Reproduced with permission.^[^ ^32^ ^]^ Copyright 2013, American Chemical Society. C) Compared to (B), further increase in throughput is achieved by using shorter microchambers (≈0.48 mm), and enhancement in detection throughput is achieved by combining spatial multiplexing and spectral multiplexing. Adapted with permission.^[^ ^35^ ^]^ Copyright 2019, Wiley. D) Assembly of graphene oxide quantum dot (GOQD) biocompatible materials on microchambers to further improve the isolation efficiency and cell activity. Reproduced with permission.^[^ ^37^ ^]^ Copyright 2022, Wiley.

Among the many types of microfluidic chips, microchamber chips combined with the single‐cell printing method that we designed can achieve high‐activity isolation and culture of single cells. The microchamber chip was placed facing upward, and the cell solution was printed onto each column in an up‐and‐down moving manner, with single cells falling into each chamber with a Poisson distribution probability under the action of gravity, allowing gentle single‐cell isolation (**Figure** **1**A). With the help of soft lithography,^[^ ^31^ ^]^ arrays of thousands of microchambers have been integrated on extremely small chips, further improving the single‐cell throughput. The combined antibody microbarcode substrate was used to detect the multiplexed factors of single cells in the microchambers, which are widely used for single‐cell isolation and multi‐factor analysis. This technique combines spatial and spectral encoding to detect 14 types of immune effector proteins at the single‐cell level, providing a new perspective for a deeper understanding of cell function and immune monitoring (Figure 1B).^[^ ^32^ ^]^ A large number of cells are isolated by the chambers to form single‐cell microchambers, and the size and shape of the microchambers can be redesigned according to the cell type and purpose of analysis.^[^ ^33^, ^34^ ^]^ Depending on the number of detection factors required, the throughput can be further increased at the expense of chip or microchamber size without affecting the detection performance (Figure 1C).^[^ ^35^ ^]^ Regardless of how it varies, the size of the microchamber is still much larger than the size of the cell, which allows it to satisfy the growth space and nutrient requirements for single cells.^[^ ^34^, ^36^, ^37^ ^]^ In addition, this space is suitable for the multiplex downstream analysis of target molecules without being affected by spectral overlap.^[^ ^32^, ^34^, ^38^, ^39^, ^40^ ^]^ At the same time, the space also provides sufficient operational room for subsequent electrical analysis. Research has suggested that changes in electrical signals can be used to estimate the position and number of cells within the microchambers.^[^ ^41^ ^]^ Therefore, electrodes can be designed within microchambers for the real‐time detection of the position and number of captured cells. Because the PDMS chip material is hydrophobic, the single‐cell separation efficiency and activity can be further improved by increasing the surface hydrophilicity through plasma or material assembly. Researchers developed a high‐throughput, live, single‐cell, multi‐marker secretory biomarker analysis platform that combines microfluidic technology with machine learning. This platform, utilizing microchamber chips assembled with graphene oxide quantum dots (GOQDs), not only ensures high‐viability culture of single cells, but also achieves high‐precision classification of tumor cells (Figure 1D).^[^ ^37^ ^]^ The study indicates that this approach has broad implications for cancer research and biomedical studies. However, the cells separated by this chip were not separated according to cell size; the separated cells were random, and the single‐cell separation efficiency was limited by the Poisson distribution.^[^ ^37^ ^]^ Some studies have used a unique method to combine droplet and valve microfluidics with microchamber array chips to surpass the Poisson distribution, which significantly improves the efficiency of single‐cell isolation and reduces cell loss. However, because this method involves assembling chips with three different structures together, it increases the complexity of the chip and reduces throughput, although it is suitable for the separation of rare cells.^[^ ^42^ ^]^

Generally, the disadvantages of low efficiency caused by a simple microchamber structure and Poisson distribution are far outweighed by the advantages of high activity, throughput, and space. Single‐cell separation activity and efficiency can be further improved by assembling biocompatible materials, and the space is sufficient for the laying of spatial barcodes, which are suitable for separations that require cell activity, especially adherent cell activity. Therefore, the most important aspect of this method is that it can achieve the in situ culture of single cells for subsequent detection. Thousands of single cells were cultured and analyzed in situ on this chip developed by our team, revealing the impact of nanomaterials and external force stimulation on the growth and differentiation of single cells during the culture process.^[^ ^43^, ^44^, ^45^ ^]^ A Microchamer chip was also used to study the grouping mechanism of single cells, enabling cancer cell subtype diagnosis and evaluation of immune cell efficacy.^[^ ^37^, ^46^, ^47^ ^]^

### 2.2 High‐Efficiency and Low‐Throughput Double‐Layer Valve Single‐Cell Isolation Chip

**Figure 2:**
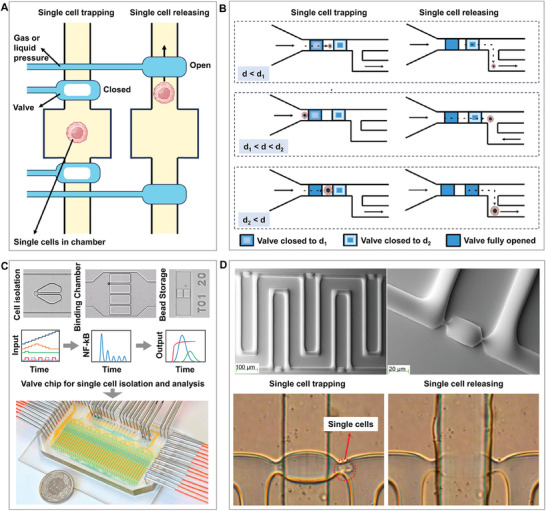
High‐efficiency and low‐throughput double‐layer valve chip. A) Methods and principles of single‐cell trapping and releasing based on single‐cell microvalve chips. B) Screening, capture, and release of single cells in different size ranges provide a controllable and precise operation platform for single‐cell research. Reproduced with permission.^[^ ^48^ ^]^ Copyright 2021, Elsevier. C) An integrated valve chip for single cell isolation, culture, and analysis to deeply understand the dynamic responses of individual cells to complex immune inputs. Reproduced with permission.^[^ ^49^ ^]^ Copyright 2016, Cell Press. D) An integrated valve chip for single‐cell isolation, screening, and recovery supports long‐term monitoring and super‐resolution imaging analysis of single cells. Adapted with permission.^[^ ^50^ ^]^ Copyright 2016, Elsevier.

A double‐layer valve chip is another chip that can achieve a high‐activity single‐cell isolation. It consisted of valves and flow channel layers. In the flow channel layer, the width, depth, and geometry of the microchannel are designed based on the cell density and cell characteristics to ensure a cell flow rate such that they can enter the chamber evenly. In the valve layer, gas or liquid is used to precisely control the opening and closing of the valve to isolate single cells in the chamber to achieve single‐cell separation. (**Figure** **2**A) This is useful for single‐cell isolation and subsequent analysis, which requires a high degree of automation, precision, and cell activity. Some studies have screened, captured, and released single cells in different size ranges by controlling microvalves that do not completely close the flow channel layer. They are directly printed onto a standard microplate, providing an efficient and accurate tool for single‐cell analysis (Figure 2B).^[^ ^48^ ^]^ In some studies, automated microfluidic valves have been used to provide precisely defined dynamic inputs to single living macrophages in 40 chambers while simultaneously applying dynamic stimuli and enabling reliable isolation, culture, and subsequent multiplex key immune parameter analyses of multiple cell types, including adherent cells (Figure 2C).^[^ ^49^ ^]^ The unique aspect of this system is its ability to reveal the time‐dependent responses of individual cells to dynamic inflammatory inputs, thus providing new tools for understanding and modeling cellular systems. Studies have also used a valve switch to isolate, screen, and recover cells with an isolation efficiency greater than 95%. Using this type of chip and a high‐resolution imaging system, a single mouse embryonic stem cell was selectively isolated, screened, and recovered without affecting the cell activity (Figure 2D).^[^ ^50^ ^]^ A study utilized enhanced micro electrical impedance spectroscopy (µEIS) technology with a membrane actuator valve, achieving a 90% single‐cell capture rate for precise characterization of age‐related impedance changes in zebrafish vascular endothelial cells. By combining a membrane actuator valve and barriers, the system efficiently captures and stabilizes single cells in the sensing area, thereby distinguishing cells at different aging stages without labeling.^[^ ^51^ ^]^ This technique demonstrates the potential of evaluating cellular states through physical parameters, providing a new perspective and tool for cell aging research.

In general, the low throughput caused by complex valve structures is often insufficient to meet the current requirements of target molecule detection, which requires big data analysis. Importantly, however, it is ideal for the single‐cell isolation of rare cells because of its high separation efficiency, good cell viability, and sufficient space for detection.

### 2.3 High‐Throughput and High‐Efficiency Microdroplet Single‐Cell Isolation Chip

**Figure 3:**
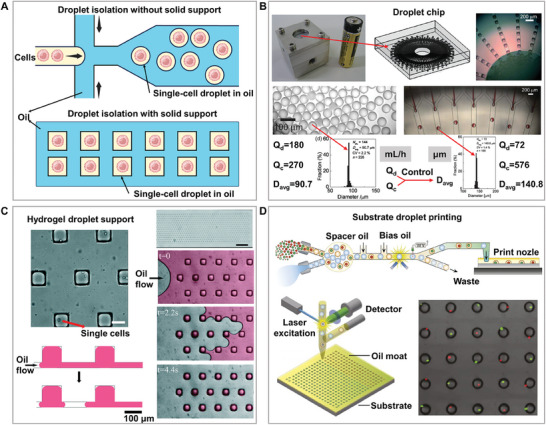
High‐throughput and high‐efficiency microdroplet chip. A) Methods and principles of solid support and support‐free single‐cell separation droplet chips. Single cells exist in individual droplets through an oil‐encapsulating liquid method. B) Large‐scale high‐throughput droplet generation based on microdroplet chips, with droplet size controlled by the flow rates of the dispersed (Q_d_) and continuous (Q_c_) phases. This breakthrough overcomes the production throughput limitations of traditional single microfluidic droplet generators, offering new possibilities for high‐throughput droplet production. Adapted with permission.^[^ ^52^ ^]^ Copyright 2012, Royal Society of Chemistry. C) Hydrogel droplet support: Encapsulation and culture of bacterial cells in anchored microdroplets highlight the versatility and potential applications of droplet chips for bioanalysis. Adapted with permission.^[^ ^58^ ^]^ Copyright 2016, Royal Society of Chemistry. D) Substrate droplet printing: High‐throughput generation of complex arrays of droplets, cells, and microparticles. This technique offers new possibilities and flexibility for the construction of single‐cell and complex assays. Adapted with permission.^[^ ^59^ ^]^ Copyright 2017, National Academy of Sciences.

The droplet microfluidic chip utilizes interlaced oil‐water channels to generate droplets to wrap and separate single cells and is considered to be one of the most promising single‐cell separation and analysis platforms. Presently, it is mainly developed into two modes: one without a solid support and the other with a solid support. (**Figure** **3**A) The probability of a droplet containing a single cell was controlled by adjusting the cell concentration, suspension medium density, and flow rate. Compared with microchambers and valve chips, the greatest advantage is the enormous throughput of thousands of single cells per second. Some studies have proposed microfluidic modules for the large‐scale production of single and composite droplets with controllable sizes and compositions, enabling higher‐throughput analysis and offering a viable solution for industrial‐scale production of droplets (Figure 3B).^[^ ^52^ ^]^ Encapsulation followed Poisson statistics, and the encapsulation efficiency was ≈ 10%−30%. Some studies have overcome the Poisson distribution using high‐aspect‐ratio microchannels and automatic sorting machine learning algorithms, thereby achieving single‐cell isolation of more than 80%.^[^ ^53^, ^54^ ^]^ For the isolation of single droplets with different cell numbers, researchers have developed a droplet microfluidic technology using solenoid valves and signal processing systems to achieve droplet sorting based on the number of cells, providing a high‐throughput platform for the separation of different cell numbers.^[^ ^55^ ^]^ However, the tradeoff between these advantages has certain disadvantages. To improve throughput and efficiency, a high initial cell concentration and small droplet size are often required.^[^ ^52^ ^]^

A high cell isolation efficiency requires high‐input single‐cell suspensions. Considering the cell loss, a higher single‐cell input suspension is often required to ensure isolation efficiency. A recent study innovatively used the DisCo method to achieve continuous processing of low‐input single‐cell suspensions at a high capture efficiency (>70%) and speeds of up to 350 cells per hour.^[^ ^56^ ^]^ Cells are encapsulated in droplets, and although studies have shown that U937 cells can maintain >80% activity for up to four days after droplet encapsulation, cell proliferation is limited because the density of single cells in a pL droplet creates a high‐density environment that prevents cell growth.^[^ ^57^ ^]^ In addition, shear stress from cell isolation and cell‐droplet surfactant interactions can also affect cell activity,^[^ ^57^ ^]^ and droplet separation presents challenges for the cell activity of some adherent or semi‐adherent cells that rely on solid growth. In some studies, cells were separated into gel droplets to provide a solid support for maintaining normal cell morphology. This platform has realized the core functionalities of microplates, including encapsulation, real‐time monitoring, and selective extraction of microdroplet content. This study demonstrated the potential application of this platform in bacteriological research by encapsulating and culturing thousands of bacterial cells in a 2D array (Figure 3C).^[^ ^58^ ^]^ In addition, droplets can be printed onto a substrate to provide solid support. This technique utilizes a fluorescence‐activated droplet sorter and a specially designed substrate to effectively construct complex arrays of droplets, cells, and microparticles at high throughput, offering a new solution for single‐cell analysis and diverse assays (Figure 3D).^[^ ^59^ ^]^ However, owing to challenges, such as shear forces generated by droplet formation,^[^ ^57^ ^]^ small droplet space,^[^ ^52^, ^60^ ^]^ biotoxicity in encapsulated droplets,^[^ ^61^, ^62^ ^]^ and the inability to effectively exchange fluids, the prospects for long‐term cell activity and normal cell growth remain to be studied, posing challenges to the isolation of high‐activity cells using droplet microfluidic chips.

In general, droplet microfluidics have excellent throughput and efficiency indicators; however, their cell activity and spatial indicators are low, limiting their application range. In addition, some studies have reported that the oil used for cell separation may have an adsorption effect on small molecules, such as proteins^[^ ^63^ ^]^ causing the loss of biological information. For applications in fluorescence‐based detection and analysis, the spherical space also limits the multiplicity of target molecule detection owing to the problem of spectral overlap.^[^ ^64^ ^]^ In addition to the optical analysis of single cells, droplet microfluidics can also incorporate electrical methods for label‐free detection of single cells. Microfluidic impedance cytometry was introduced to classify single cells by detecting changes in electrical impedance as the cells passed through a series of microelectrodes.^[^ ^65^ ^]^ Some research have distinguished cell activity based on changes in impedance.^[^ ^66^ ^]^ Compared to optical methods, it offers advantages, such as no need for labeling and the capability for real‐time monitoring, providing unique benefits in applications where biomarker detection is not required.

### 2.4 High‐Throughput and High‐Efficiency Microwell Single‐Cell Isolation Chip

**Figure 4:**
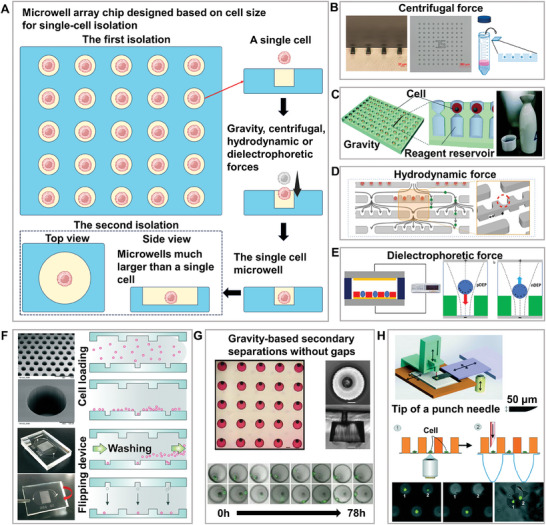
High‐throughput and high‐efficiency microwell chip. A) Methods and principles of the first and second isolation of single cells based on a single‐cell microwell chip. The forces involved in separation include gravity, centrifugal force, hydrodynamic force, and dielectrophoretic force. Microwells used for the second separation are usually larger than those used for the first isolation. B) Separation based on centrifugal force. Adapted with permission.^[^ ^67^ ^]^ Copyright 2015, American Chemical Society. C) Separation based on gravity. Adapted with permission.^[^ ^68^ ^]^ Copyright 2017, Royal Society of Chemistry. D) Separation based on hydrodynamic force. Reproduced with permission.^[^ ^70^ ^]^ Copyright 2013, National Academy of Sciences. E) Separation based on dielectrophoretic force. Adapted with permission.^[^ ^77^ ^]^ Copyright 2019, American Chemical Society. F) Gravity‐based secondary separation with gaps. The “gap” refers to the distance between the two chips for secondary separation. Reproduced with permission.^[^ ^79^ ^]^ Copyright 2015, Royal Society of Chemistry. G) Gravity‐based secondary separations without gaps. The “gaps” refers to the distance between the two chips for secondary separation. Reproduced with permission.^[^ ^80^ ^]^ Copyright 2021, Cold Spring Harbor Laboratory. H) Punch secondary separations based on hydrodynamic force. Reproduced with permission.^[^ ^81^ ^]^ Copyright 2015, Royal Society of Chemistry.

Microwell array chips often use gravity, centrifugal force, hydrodynamic force, or dielectrophoretic force to separate single cells into traps designed according to cell size to achieve single‐cell separation. However, if subsequent analysis requires ensuring cell activity or additional cultivation space, a secondary transfer may be required. (**Figure** **4**A) During the isolation process, the cells did not enter the microwells into which single cells had entered. They continue to enter the surrounding microwells through gravity, centrifugal, hydrodynamic, or dielectrophoretic forces, thereby achieving high‐throughput and efficient single‐cell isolation. Some studies have used centrifugal force and truncated cone‐shaped microwell structures to achieve a single‐cell separation efficiency of 90% and a single‐cell throughput of over 10000 on a chip of 1 square centimeter within a few seconds, realizing efficient and high‐throughput real‐time observation of single‐cell apoptosis. This demonstrates the great potential of the microwell chip technology in revealing cellular heterogeneity and drug responses at the single‐cell level (Figure 4B).^[^ ^67^ ^]^ Some studies has used gravity for single‐cell separation, and a technique called one‐step micromolding has been developed. This approach successfully produced a structure similar to the shape of a Japanese sake vessel, aimed at increasing the reaction volume of microwells for related applications,^[^ ^68^ ^]^ such as reverse transcription‐polymerase chain reaction or genome amplification (Figure 4C). Some studies separated individual MCF‐7 cells into a microwell array using gravity, isolated them with oil to prevent crosstalk, and employed dual‐nanopore technology for the electrical detection of secreted reactive oxygen species.^[^ ^69^ ^]^ This approach offers high sensitivity and selectivity for single‐cell electrical analysis, and provides a new method for the development of future biosensors. With the aid of hydrodynamics, the multistage separation and screening microfluidic chip uses traps with holes of different sizes at the bottom, providing 40, 30, 20, and 15 µm 4‐level layer‐by‐layer filtration. Cancer cells in the ascites were isolated using correspondingly sized holes in each layer for subsequent fluorescent analysis of cell surface biomarkers, providing a new monitoring method for cytotoxic and molecular targeted therapy in clinical trials for ovarian cancer (Figure 4D).^[^ ^70^ ^]^ Similarly, based on hydrodynamic microwell isolation, researchers demonstrated that µEIS can be used to precisely measure the impedance of single cells without relying on fluorescent labeling.^[^ ^71^, ^72^, ^73^, ^74^ ^]^ Some studies have effectively measured single‐cell impedance by combining improved microfluidic technology with µEIS. This work not only expanded the frequency detection range, but also successfully distinguished the different states of mouse oocytes, demonstrating the potential of this technology for label‐free cell analysis.^[^ ^71^ ^]^ Another study integrated impedance flow cytometry and impedance spectroscopy techniques to effectively and passively capture single cells and analyze their electrical properties, showcasing their potential for identifying and quantifying the impedance characteristics of cancer cells.^[^ ^72^ ^]^ These label‐free impedance measurement methods provide us with a tool that complements traditional fluorescence analysis, enabling us to analyze and understand the biophysical properties of cells from a broader perspective. Several studies have successfully separated target cells with up to 90% efficiency for single‐cell isolation using magnetic.^[^ ^75^ ^]^ or dielectrophoretic forces.^[^ ^76^ ^]^ Furthermore, some studies have captured single PC‐3 cells in micropores using dielectrophoresis, and then applied µEIS microfluidic technology for drug treatment with 100 µM enzalutamide, monitoring the cell death process through impedance measurement (Figure 4E).^[^ ^77^ ^]^

Compared to the use of gravity, centrifugal force, or fluidic force, dielectrophoresis isolation offers the unique advantages of high selectivity, sensitivity, and gentle processing, making it highly suitable for the isolation of rare or small‐sized biological particles. Exosomes are nanoscale membrane vesicles that are particularly suitable for isolation via dielectrophoresis. Researchers have developed methods for exosome isolation and size classification based on direct current insulator‐based dielectrophoresis microfluidic technology.^[^ ^78^ ^]^ Through specially designed microchannels and the application of 2000 V, effective capture and sorting of exosomes were achieved. The successful application of this technology provides a new direction for the development of efficient microfluidic devices, particularly for the rapid separation and characteristic analysis of exosomes. Theoretically, this technology could be applied to exosomes secreted by single cells. Further application of exosomes secreted by single cells to explore the heterogeneity of differently sized exosomes secreted by the same cell could offer new insights.

Although the above methods are relatively gentle for cell separation, the normal growth and proliferation of cells may be affected by the small spaces of the microwells, and other subsequent applications that require long‐term cell activity often require a second high‐activity transfer. Some studies have proposed methods for separating cells a second time to further improve their long‐term activity. First, high‐throughput and high‐efficiency single‐cell microwell separation was performed by gravity, and then the chip was flipped over to transfer single cells from small to large wells for culture and observation. The method is easy to perform, and not only improves the efficiency and throughput of single‐cell separation but also ensures the normal growth and proliferation of cells. However, the non‐enclosed single‐cell space caused by the gap between the large and small wells during the secondary separation process causes crosstalk of the detection signals, and the integrated design of the two chips is not easily expanded to subsequent applications (Figure 4F).^[^ ^79^ ^]^ Therefore, a method was developed for capturing single cells by gravity using funnel‐shaped micropores. The small holes in the top layer were used to separate the cells according to their size, and the cells were trapped in the large holes in the bottom layer, where the space was sufficient to ensure the normal growth and proliferation of suspended or adherent cells. Furthermore, through the unique design of the narrow‐mouth micropore arrays, the positional stability of single cells during repeated detection processes was efficiently maintained, thereby reducing the cell loss rate to below 3%. However, there was a possibility of multiple cells, and the single‐cell throughput and capture efficiency decreased (Figure 4G).^[^ ^80^ ^]^ Another type of microwell array chip uses hydrodynamic force to separate cells. Owing to the slightly larger microwells, the single‐cell efficiency decreased to 70%. Secondary screening of single cells by punching improves the recovery rate of target cells, broadening the application range; however, because the cells are transferred to a well plate, subsequent applications may encounter the problem of high consumption of reagents and contamination in traditional analysis (Figure 4H).^[^ ^81^ ^]^

In general, the microwell chip technology completely overcomes the Poisson distribution and greatly improves the throughput and efficiency of single‐cell separation. To ensure the normal growth and proliferation activity of cells, especially adherent cells, it is necessary to increase the size of the pores. As a result, the throughput and efficiency are reduced, but the Poisson distribution can still be overcome to a certain extent with the help of special structures and hydrodynamic forces.^[^ ^81^ ^]^ To ensure the four quality indicators of throughput, efficiency, activity, and space at the same time, it is necessary to perform secondary screening and separation. Although secondary separation can separate target cells into well plates or large wells, it is the same as droplet microfluidic chips in terms of performing fluorescence detection before secondary screening because the process lacks detection space and is affected by spectral overlap.^[^ ^82^ ^]^ Electrical detection does not consider spectral overlap. Because electrical detection requires single cells to remain in a fixed position for analysis, microwell chips may be the most suitable single‐cell isolation chip technology for electrical signal analysis. A comparison of the quality indicators, such as throughput, efficiency, space, and cell activity, of the above four single‐cell isolation chip technologies is shown in **Table** **2**.

**Table 2:** . Comparison of the isolation quality of various single‐cell isolation chip technologies.

Biochip technology	Throughput	Efficiency	Space	Cell activity
Microchamber	High	<37%	High	High
Valve	Low	100%	High	High
Droplet	High	<90%	Medium	Medium
Microwell	High	>70%	Low	Medium

## 3 Applications of the Single‐Cell Isolation Chip for Single‐Cell Multiomic and Exosome Analysis

Single‐cell isolation is a prerequisite for downstream single‐cell analysis applications, and the quality of single‐cell isolation determines the accuracy of the target molecular analysis and its application range. Traditional single‐cell separation methods have certain application limitations because of poor separation environments and low throughput, which have been discussed in detail in other related reports. Here, we discuss the genomics, epigenomics, transcriptomics, proteomics, and emerging exosome analysis applications based on single‐cell isolation chips in different scientific fields. In general, genomics, epigenomics, and transcriptomics applications usually require single cells to be fixed, permeabilized, or lysed after isolation and do not require high‐activity single‐cell isolation, which can further reduce the separation space and improve throughput and efficiency. Proteomics and exosome analysis often need to ensure cell activity and thus require high‐activity single‐cell isolation, which in turn requires a certain amount of throughput and efficiency to improve the separation space. Monoclonal drug screening is a special area of proteomics research. As target cells are relatively rare and precious, high‐efficiency isolation is often required prior to high‐activity isolation to reduce cell loss.

### 3.1 Application of the Single‐Cell Isolation Chip for Single‐Cell Genome Analysis

Genetic mutations and variations may be inherited or may arise during cellular proliferation and differentiation. Traditional genomic studies predominantly use homogeneous samples, and genetic heterogeneity and functional diversity among individual cells have been overlooked. The emerging field of single‐cell genomics provides a comprehensive analysis of the genome at the individual cell level, enabling the characterization of authentic cellular features and functions. However, the low initial genome concentration in single cells presents a significant challenge in single‐cell genomics. Thus, a pivotal step in single‐cell genomics analysis is to amplify picogram (pg) or nanogram (ng) quantities of DNA to microgram (µg) levels, meeting the sequencing requirements. Low starting genome concentrations may impede amplification efficiency, potentially introducing biases or errors and compromising the overall data quality. Limitations persist in many traditional methods, such as low throughput, high cost, difficulty of use, limited cell purity, and limited capture efficiency, which restrict their applicability in large‐scale and high‐throughput studies. Advancements in microfluidic chip technology have spurred rapid developments in genomic research. Researchers have continuously explored novel techniques and methods to facilitate more comprehensive single‐cell genomic analyses with the aim of accurately discerning cell types and states. However, the need for high‐throughput single‐cell genomic analyses poses a challenge for on‐chip single‐cell isolation.

**Figure 5:**
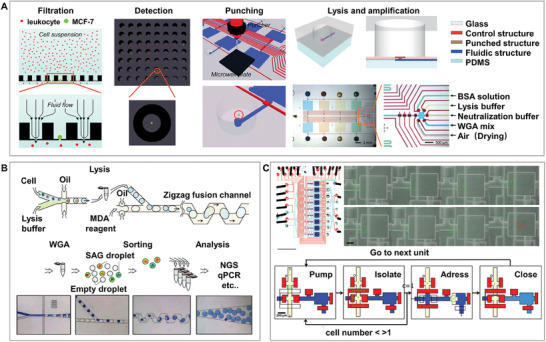
Single‐cell isolation chip for genomics. A) Integration of single‐cell isolation, screening, lysis, and DNA amplification detection, using microwell chips offers an effective approach for advanced genetic characterization. Adapted with permission.^[^ ^83^ ^]^ Copyright 2015, Royal Society of Chemistry. B) DNA amplification and detection in droplets using passive droplet fusion, leveraging the droplet‐based approach, enables high‐throughput acquisition of contamination‐free and cell‐specific sequence reads. Adapted with permission.^[^ ^86^ ^]^ Copyright 2017, Springer Nature. C) The nanoliter volume chamber improves the DNA amplification quality compared to microliter volume utilizing the valve technology and facilitates the efficient analysis of single‐cell genomes. Adapted with permission.^[^ ^91^ ^]^ Copyright 2007, Public Library of Science.

Some studies have used microwell chips to isolate and screen MCF7 tumor cells in whole blood and punch the target cells into a microreaction chamber for lysis and DNA amplification analysis (**Figure** **5**A).^[^ ^83^ ^]^ The input end of the micro‐reaction chamber consists of a valve structure that can sequentially pump the cell lysis and DNA amplification reagents. Owing to the small volume of the micro‐reaction chamber, reagent waste is reduced, and the amplification efficiency and detection sensitivity are improved. Although microwell isolation chips provide sufficient throughput and separation efficiency, the throughput of single‐cell analysis is still insufficient for big data analysis of genes because on‐chip DNA amplification and detection still rely on the valve structure. However, in terms of drug development, there are some studies that can lyse cells in situ in microwells and reveal the degree of DNA damage caused by drugs in cells through fluorescence detection; throughput and efficiency have been greatly improved.^[^ ^84^ ^]^ Compared with fluorescence detection, in terms of electrical detection, researchers have showcased an advanced method for detecting single‐molecule DNA within a single cell using 3D integrated nanopore technology, achieving efficient lysis and direct detection of DNA molecules at the single‐cell level. This method not only demonstrates the changes in electrical signals as millimeter‐long polynucleotides pass through nanopores, but also opens new possibilities for single‐molecule sequencing and multiomics analysis at the single‐cell level, paving a new path for single‐cell analysis on a chip.^[^ ^85^ ^]^ There is a potential for combining this with micropore array microfluidic chip technology to further increase the detection throughput.

As mentioned above, because DNA amplification and detection require on‐chip blocking, droplet chips are powerful tools for large‐scale DNA analysis. Some studies have used passive droplet fusion to add lysates or WGA reagents to the droplets after isolating the cells using droplet chips. Because individual cells were isolated in small compartments, their genomes could be amplified to saturation without contamination (Figure 5B).^[^ ^86^ ^]^ This contamination‐free single‐cell DNA amplification method enables high‐quality, high‐throughput DNA analysis. Therefore, sequence acquisition was possible. Single‐cell genome research based on droplet chips has been extensively conducted and applied in microbiology,^[^ ^87^, ^89^ ^]^ and immunology.^[^ ^90^ ^]^

The integration of the valve chip itself can reduce the possibility of foreign DNA and cross‐chamber contamination and is also commonly used in genomics research. Researchers have developed new valve microfluidic chip methods to improve the efficiency and quality of amplified genomes from single cells. By conducting the MDA reaction at the nanoliter level, nonspecific synthesis was significantly reduced, making this technique potentially transformative for single‐cell genomic research (Figure 5C).^[^ ^91^ ^]^ In addition, valves are commonly used to integrate multistep single‐cell reactions, and valve chips are widely used in cancer research,^[^ ^92^, ^93^ ^]^ drug therapy,^[^ ^94^ ^]^ and microbiology,^[^ ^91^ ^]^ but Valve‐based methods typically have low throughput and require significant human time.^[^ ^91^, ^92^ ^]^

We summarized and divided the current research areas for single‐cell isolation based on microwells, droplets, and valve chips in single‐cell genome analysis, as shown in **Table** **3**. Genomics can reveal complex cellular characteristics and functions that are important for understanding single‐cell functions. The need to analyze large amounts of single‐cell data to study complex cell functions presents certain requirements for throughput and efficiency of single‐cell isolation. In addition, because genomic analysis usually requires cells to be fixed, permeabilized, or even lysed immediately after single‐cell isolation, it does not require much cell activity, and the small separation space can reduce the consumption of reagents and improve reaction efficiency. Therefore, microwell‐based and droplet‐based chips are the most suitable separation technologies for single‐cell genome applications.

**Table 3:** Use of single‐cell isolation chip technology for genome analysis in different fields.

Research field	Chip structure	Detection object	Detection information	Year	Reference
Cancer research	Microwell	MCF7 tumor cells	DNA	2015	[83]
Cancer research	Valve	ER^−^/PR^−^/HER2^+^ breast cancer cells	DNA	2015	[92]
Cancer research	Valve	LS174T colorectal cancer cells	DNA	2018	[93]
Drug treatment	Valve	Lung cancer cells	DNA	2018	[94]
Drug screening	Droplet	Glioma cells	DNA	2016	[84]
Immunology	Droplet	Lymphoblasts	DNA	2018	[90]
Microbiology	Droplet	Bacteria	DNA	2019	[89]
Microbiology	Valve	Escherichia coli	DNA	2007	[91]
Microbiology	Droplet	Bacterial and human cancer cells	DNA	2017	[86]
Microbiology	Droplet	Microalgal and yeast cells	DNA and RNA	2017	[87]
Microbiology	Droplet	Red sea environmental sample	DNA	2021	[88]

### 3.2 Application of the Single‐Cell Isolation Chip for Single‐Cell Epigenomic Analysis

Although the genome is often seen as the blueprint of life, different cell types read this blueprint in unique ways, activating only a portion of the potential genes. Cell‐specific gene activity is closely regulated by epigenomics and involves DNA methylation,^[^ ^95^, ^96^, ^97^ ^]^ histone modifications,^[^ ^98^, ^99^, ^100^ ^]^ and chromatin accessibility.^[^ ^101^, ^102^, ^103^, ^104^, ^105^ ^]^ Traditional epigenomic research methods often analyze by averaging the characteristics of a large number of cells, thereby ignoring heterogeneity at the single‐cell level. Single‐cell epigenomic analysis allows us to understand subtle differences between different cell types or states, revealing new cell subgroups and identifying rare cell types. However, epigenomic information at the single‐cell level is scarce, and the accuracy and robustness of experimental operations and analysis techniques are in high demand and easily affected by experimental process errors. The latest microfluidic technology has significantly improved the efficiency of sample processing and detection sensitivity of single‐cell epigenomics through its flexible design and adjustable parameters, opening new avenues for finely distinguishing different cell types and states. However, similar to genomic research, single‐cell epigenomics poses challenges to the technology of single‐cell isolation‐on‐chips because of the demand for high‐throughput analysis.

Single‐cell sequencing assay for transposase‐accessible chromatin (scATAC‐seq), single‐cell bisulfite sequencing, and single‐cell chromatin immunoprecipitation sequencing (scChIP‐seq) are powerful technologies that reveal complex epigenomic regulatory networks within cells from different perspectives. Together, these techniques provide a comprehensive framework for understanding the dynamics and complexity of gene expression regulation at the single‐cell level.

**Figure 6:**
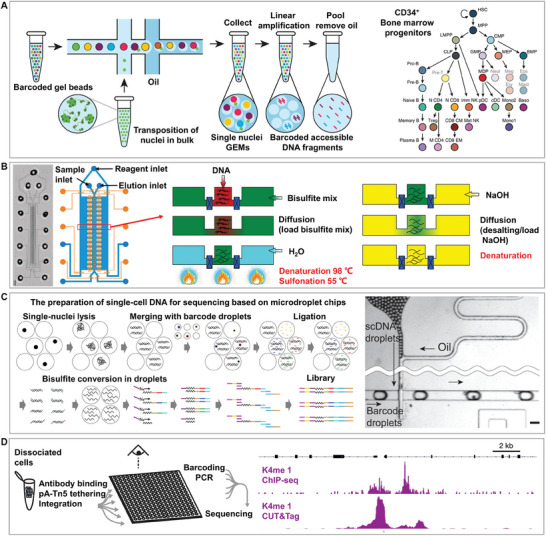
Single‐cell isolation chip for epigenomics. A) Single‐cell sequencing assay for transposase‐accessible chromatin (scATAC‐seq) on microdroplet chips facilitates the identification of transcriptional elements and cell differentiation in over 200000 human immune cells within tumor environments. Adapted with permission.^[^ ^101^ ^]^ Copyright 2019, Springer Nature. B) Valve microfluidic technology with microfluidic diffusion‐based reduced representation bisulfite sequencing (MID‐RRBS) offers low‐DNA high‐efficiency epigenomic insights. Adapted with permission.^[^ ^97^ ^]^ Copyright 2018, Springer Nature. C) Drop‐BS used droplet‐based bisulfite sequencing for scalable single‐cell DNA methylation analysis, processing up to 10000 samples in two days, thereby significantly advancing epigenomics. Adapted with permission.^[^ ^95^ ^]^ Copyright 2023, Springer Nature. D) CUT&Tag on microwell chips marks a leap in single‐cell epigenomics by targeting histone modifications for live‐cell sequencing, offering a simpler lower‐reagent workflow than single‐cell chromatin immunoprecipitation sequencing (scChIP‐seq) and boosting single‐cell precision and throughput. Adapted with permission.^[^ ^100^ ^]^ Copyright 2019, Springer Nature.

scATAC‐seq technology assesses chromatin openness or accessibility at the single‐cell level. A recent study analyzed the development of human immune cells and the state of T cell exhaustion within the tumor microenvironment at a single‐cell resolution. Specifically, using massively parallel scATAC‐seq technology on a droplet‐based platform, this study demonstrated the powerful capability of the platform to reveal cell‐type‐specific transcriptional regulatory elements, disease‐associated enhancer activities, and cell differentiation trajectories. Leveraging droplet microfluidics greatly enhanced cell throughput by analyzing data from over 200000 single cells (**Figure** **6**A).^[^ ^101^ ^]^ Single‐cell bisulfite sequencing focuses on DNA methylation at the single‐cell level. For the first time, a recent study utilized valve microfluidic technology with a low‐input microfluidic diffusion‐based reduced representation bisulfite sequencing (MID‐RRBS) method to precisely analyze single‐cell DNA methylation states in the mouse brain. This revealed methylation patterns in neurons and glial cells, along with the cell‐specific effects of atypical antipsychotic drugs, thus providing a new tool for understanding epigenomic regulation in the nervous system. This approach offers several advantages over traditional methods, including less DNA requirement and more efficient bisulfite conversion, which is critical for studies with limited samples, owing to the integrated and multistep capabilities of valve chips (Figure 6B).^[^ ^97^ ^]^ As mentioned previously, valve chips are limited by their separation throughput, which prevents the processing of a large number of single cells in one experiment. Scientists have developed an innovative technology, called Drop‐BS, which is a droplet‐based bisulfite sequencing method for high‐throughput analysis of single‐cell DNA methylomes. This technique overcomes the limitations of traditional single‐cell methylation sequencing by processing up to 10000 single‐cell samples within two days, significantly enhancing the efficiency and scale of single‐cell DNA methylation studies and marking a significant advance in the field of epigenomics (Figure 6C).^[^ ^95^ ^]^ One of the notable advantages of nanopore sequencing technology is its ability to perform electrical detection without the need for PCR amplification, and it can detect specific chemical modifications, such as DNA methylation.^[^ ^106^ ^]^ or chromatin accessibility.^[^ ^107^ ^]^ This makes nanopore sequencing is a flexible and powerful tool for epigenetic research. However, current research focuses on single molecules and does not effectively integrate single‐cell isolation chip technologies. The combination of nanopore sequencing with single‐cell chip technology for single‐cell epigenomics is promising. The scChIP‐seq focuses on histone modifications at the single‐cell level. Research has developed a CUT&Tag technique applicable to microwell chips, making a breakthrough in single‐cell epigenomic analysis. Sequencing libraries were generated directly from live cells by targeting specific histone modifications with the A‐Tn5 transposase fusion protein. Compared to traditional scChIP‐seq, CUT&Tag simplifies the workflow, reduces reagent use, and is compatible with microwell chips, thereby enhancing the precision and high‐throughput analysis capabilities of single‐cell operations. This technology optimizes the acquisition of epigenomic data and provides a new tool for understanding cellular heterogeneity and disease mechanisms (Figure 6D).^[^ ^100^ ^]^

We summarized and divided the current research areas for single‐cell isolation based on microwells, droplets, and valve chips in single‐cell epigenome analysis, as shown in **Table** **4**. Epigenomics reveals how genetic information can be regulated through chemical modifications without altering the DNA sequence, which is crucial for understanding the functions of individual cells. Because epigenomic analysis often requires modifications or measurements of DNA and proteins in cells, it poses higher demands for the resolution and operational flexibility of single‐cell isolation technology. Microwell‐based chips are commonly used for fixing and processing large numbers of single‐cell samples because of their ability to achieve high‐density cell capture and efficient spatial utilization, thereby reducing reagent consumption and improving reaction efficiency. However, open microwells can cause contamination and crosstalk. Droplet‐based chips, with their unique ability for the high‐throughput encapsulation of single cells and control of the microenvironment, are particularly suitable for epigenomic analyses that require efficient conversion and broad genomic coverage, such as DNA methylation or chromatin accessibility assays. Valve chip technology, with its precise fluid control capability, allows for fine chemical processing at the single‐cell level, which is suitable for epigenomic research that requires precise control of reaction conditions. However, its main drawback is its slightly lower throughput. Therefore, microwell‐based and droplet‐based chips are currently the most suitable isolation technologies for single‐cell epigenetic applications.

**Table 4:** . Use of single‐cell isolation chip technology for epigenome analysis in different fields.

Research field	Chip structure	Detection object	Detection information	Year	Reference
Cancer research	Droplet	Human blood and basal cell carcinoma	Chromatin accessibility	2019	[101]
Cancer research	Droplet	Mixed cell lines, mouse and human brain tissues	DNA methylation	2023	[95]
Immunology	Droplet	Human bone marrow‐derived cells	Chromatin accessibility	2019	[102]
Immunology	Droplet	Human hematopoietic cells	Chromatin accessibility	2021	[103]
Immunology	Droplet	B cells	DNA methylation	2022	[96]
Immunology	Microwell	Peripheral blood mononuclear cells	Chromatin accessibility	2018	[104]
Neuroscience	Droplet	Mouse brain cells	Histone modifications	2021	[98]
Neuroscience	Droplet	Mouse and drosophila cells	Chromatin accessibility	2022	[105]
Neuroscience	Valve	Mouse cerebellum cells	DNA methylation	2018	[97]
Embryology	Droplet	ES cells, fibroblasts, and hematopoietic progenitors	Histone modifications	2015	[99]
Cell classification	Microwell	K562 and H1 cells	Histone modifications	2019	[100]

### 3.3 Application of the Single‐Cell Isolation Chip for Single‐Cell Transcriptome Analysis

The genome and epigenome provide fundamental instructions and regulatory mechanisms that determine how genetic information is potentially expressed. Each cell type makes different uses of the genome, expressing a subset of all possible genes. This variation has motivated efforts to characterize the molecular composition of various cell types in humans and multiple model organisms using both transcriptional and proteomic approaches.^[^ ^108^ ^]^ The transcriptome comprises a complete range of mRNAs. The mRNA profile of a single cell changes rapidly with changes in the internal and external cellular environments. These mRNA molecules provide useful information about many cell states and types. To gain insight into this information, quantification of gene expression heterogeneity in single cells is required for an in‐depth analysis of single‐cell transcriptomes. Single‐cell transcriptome analysis presents challenges at the single‐cell level due to several factors. One major challenge is the low RNA content, which can be attributed to insufficient reverse transcription and amplification efficiency, as well as the volatility and degradation of RNA molecules. In addition, the dynamic variability in gene expression levels necessitates a large amount of single‐cell transcriptome data, making data analysis more complex. This poses a challenge for the development of single‐cell isolation processes to minimize the impact of such unstable factors.

**Figure 7:**
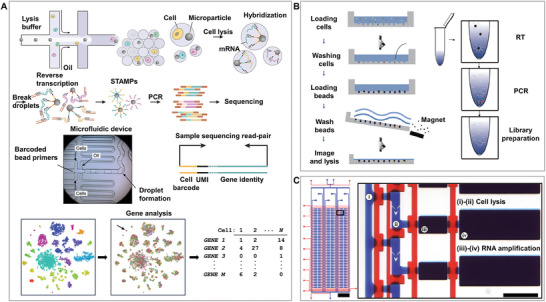
Single‐cell isolation chip for transcriptomics. A) Droplet chip technology for RNA transcription analysis facilitates the high‐throughput examination of gene expression at the single‐cell level, identifying 39 unique cell populations within the mouse retina and establishing an integrated molecular expression map. Reproduced with permission.^[^ ^116^ ^]^ Copyright 2015, Cell Press. B) Application of Microwell‐seq technology for the analysis of RNA transcription using a microwell chip, leading to the examination of over 400000 mouse single cells. Reproduced with permission,^[^ ^109^ ^]^ Copyright 2018, Cell Press. C) Valve‐based chip for cell lysis and RNA amplification facilitates high‐throughput and accurate reverse transcription‐quantitative polymerase chain reaction (RT‐qPCR) gene expression measurements across hundreds of single cells, significantly lowering the expenses while increasing the sensitivity of detection. Adapted with permission.^[^ ^121^ ^]^ Copyright 2011, National Academy of Sciences.

Microwell chips are easy to operate and widely used in single‐cell transcriptome analysis because of their excellent throughput, efficiency, and parallelism. Microwell‐seq is a low‐cost, high‐throughput platform for single‐cell RNA sequencing that enables single‐cell profiling of the transcriptome of a complex mammalian system in mice, covering more than 400000 cells in mouse organs and can accurately identify cell types through single‐cell transcriptional profiling. This platform, which uses reusable silicon and PDMS to fabricate agarose microarray chips, saves cost and time, separates single cells and barcoded mRNA‐capturing magnetic beads into microwell arrays by gravity, requires no special equipment, and is easy to use in small laboratories (**Figure** **7**B).^[^ ^109^ ^]^ Although the open space used in this method is convenient, cross‐contamination is possible. Some studies have sealed microwells with a semipermeable polycarbonate membrane, which allows solution exchange during cell lysis while still capturing biomacromolecules, ensuring better mRNA capture and minimizing cross‐contamination.^[^ ^110^ ^]^ In addition, by integrating nanopore sequencing with microwell technology, RNA molecules can be sequenced directly based on electrical signals without requiring RNA amplification, thereby reducing the possibility of signal interference. Furthermore, researchers developed a software tool called ScNapBar that enhances the accuracy of long‐read RNA sequencing using nanopore technology.^[^ ^111^ ^]^ Integrating the single‐cell microwell chip technology with novel nanopore sequencing technology is crucial for the development of future single‐cell sequencing technologies.^[^ ^112^ ^]^ In addition to being used in cell classification research, single‐cell microwell chips are also widely used in drug treatment,^[^ ^113^ ^]^ drug screening,^[^ ^114^ ^]^ and immunology^[^ ^115^ ^]^ in recent years.

Droplet‐based methods provide scalable platforms for single‐cell RNA sequencing. Cells, reagents, and barcoded detection magnetic beads were encapsulated in droplets, and cell lysis, RT, and PCR were performed. Some studies have analyzed the transcriptome of 44808 mouse retinal cells and identified 39 cell populations, including a molecular barcoding strategy that records the cellular origin of each mRNA. This barcode consisted of PCR primers, a cellular barcode for distinguishing cells, a UMI for distinguishing mRNA, and poly(T) for binding to mRNA. The enclosed space reduces the possibility of reagent contamination, but the method provides minimal control during droplet formation to ensure the isolation of single cells, resulting in inefficient encapsulation, which prevents application to rare cell samples. Moreover, the peripheral equipment (flow cytometry) is expensive, but this is still the best single‐cell transcriptome analysis platform (Figure 7A).^[^ ^116^ ^]^ In the field of electrical analysis, researchers have developed an improved droplet protocol for Chromium 10x, integrating short‐read and long‐read (nanopore) sequencing along with a new computational workflow (FLAMES). This approach has enabled the discovery of full‐length isoforms, splicing analyses, and mutation detection at the single‐cell level.^[^ ^117^ ^]^ Single‐cell droplet chips are widely used in microbiology,^[^ ^87^, ^118^ ^]^ and cell classification.^[^ ^119^ ^]^

Valve‐based methods usually have low throughput and require considerable human time; however, their integrated controllability reduces the consumption of reagents and the possibility of contamination. Researchers have developed an MID‐RNA‐seq valve microfluidic device, which simplifies the workflow through further integration, addresses the complexity and cost issues of single‐cell RNA sequencing, and offers a new, efficient, and accurate approach for transcriptomic studies of rare cell samples.^[^ ^120^ ^]^ Studies have demonstrated that isolating, sorting, and lysing cells within a closed and integrated microfluidic device can minimize the potential for contamination observed with other methods. The development of an integrated microfluidic device capable of precise gene expression measurement from hundreds of single cells was realized using high‐throughput microvalve single‐cell RT‐qPCR technology, with all preparation steps conducted in a reaction volume of 140 nL. In addition to isolation and culture, cells can be lysed on the chip, and PCR amplification can be performed for subsequent analysis (Figure 7C).^[^ ^121^ ^]^ This platform significantly enhances the throughput and precision of single‐cell gene expression analysis, providing a powerful tool for understanding gene regulatory mechanisms and disease origins at the single‐cell level. Due to the excellent integration of valve chips, they are widely used in cancer research.^[^ ^121^, ^122^, ^123^, ^124^ ^]^

We summarized and divided the current research areas for single‐cell isolation based on microwells, droplets, and valve chips in single‐cell transcriptome analysis, as shown in **Table** **5**. Transcriptomics is an important approach for studying cell phenotypes and functions. Unlike the genome, the transcriptome is affected by cell growth period, growth environment, and reactions to drugs. Although transcriptome analysis has certain requirements regarding the growth environment of cells, cells are often lysed immediately after isolation and do not require long‐term culture. In addition, the small space can reduce the possibility of contamination and improve the reaction efficiency; therefore, droplets and microwells with higher throughput and efficiency are usually selected for transcriptomics applications.

**Table 5:** Use of single‐cell isolation chip technology for transcriptome analysis in different fields.

Research field	Chip structure	Detection object	Detection information	Year	Reference
Cancer research	Valve	CTCs	RNA	2020	[122]
Cancer research	Valve	THP‐1 (ATCC TIB‐202) human monocytes	mRNA	2016	[123]
Cancer research	Valve	MCF‐7 cells	mRNA	2016	[124]
Cancer research	Valve	K562 and hESC cells	RNA	2011	[121]
Drug treatment	Microwell	A549 cells	RNA	2022	[113]
Drug screening	Microwell	Colorectal cancer cells	RNA	2023	[114]
Immunology	Microwell	APC and T cells	RNA	2023	[115]
Neuroscience	Droplet	Mouse retinal cells	RNA	2015	[116]
Cell classification	Droplet	MOLT‐4, SK‐BR3, MCF7, and U‐2 OS cells	RNA	2021	[119]
Cell classification	Microwell	Mouse cells	RNA	2018	[109]
Cell classification	Microwell	Human U87 and mouse 3T3 cells	RNA	2016	[110]
Microbiology	Droplet	Bacterial, yeast, and human cells	RNA	2023	[118]
Microbiology	Droplet	Microalgal and yeast cells	DNA and RNA	2017	[87]

### 3.4 Application of the Single‐Cell Isolation Chip for Single‐Cell Proteome Analysis

As sequencing technologies have matured, genomics, epigenomics, and transcriptomics have revealed substantial cellular heterogeneity. Although genes determine the fundamental differences between cells, cell phenotype or secretion behavior is still governed by non‐genetic factors, such as the cell state and external environmental influences. Proteins represent the main functional mechanisms of cells and usually exhibit a wide dynamic range of expression, which directly reflects the phenotypic and behavioral characteristics of cells. Compared to DNA and RNA, the chemical complexity of proteins is much higher, and the abundance of relevant functional proteins is low and cannot be directly amplified to improve the signal‐to‐noise ratio. Single‐cell proteomics can reveal the cellular phenotypic heterogeneity and cell‐specific functional networks of biological processes.^[^ ^125^ ^]^ Accurate and multiplex detection of proteomic information from a single cell is a major technical challenge that requires absolute quantification and multiplex detection. With the ongoing development of chip‐based proteomic detection technology, the sensitivity and throughput of proteomic detection have improved. However, this often requires a single‐cell isolation process before detection to separate high‐quality single cells to ensure high cell activity and sufficient detection space, which poses challenges for improving the quality of cells separated by microfluidic chips.

**Figure 8:**
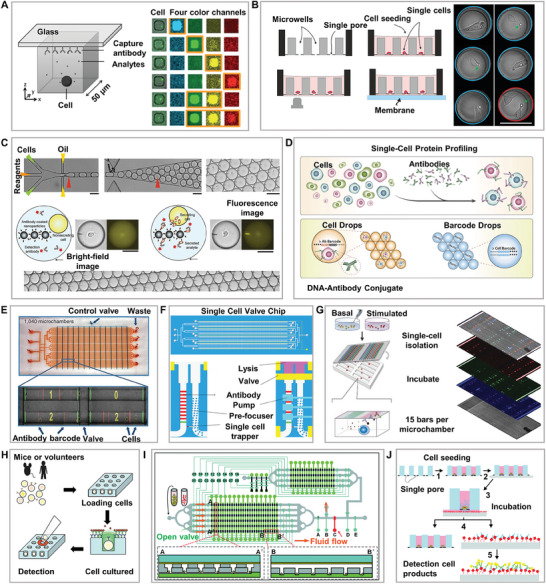
Single‐cell isolation chip for proteomics. A) Multicolor protein detection based on a microwell chip, with detection throughput affected by spectral overlap. Reproduced with permission.^[^ ^126^ ^]^ Copyright 2012, National Academy of Sciences. B) Protein dynamic detection based on a microwell chip, where the antibody membrane for protein detection can be replaced. Reproduced with permission.^[^ ^128^ ^]^ Copyright 2021, Mdpi. C) Detection of functionalized antibody magnetic beads based on droplet chips. Reproduced with permission.^[^ ^64^ ^]^ Copyright 2013, Royal Society of Chemistry. D) Detection of DNA‐antibody magnetic beads based on droplet chips. Reproduced with permission.^[^ ^133^ ^]^ Copyright 2017, Springer Nature. E) Secreted protein detection based on the valve chip, marking the first appearance of multiplex barcode chip detection for secreted proteins. Reproduced with permission.^[^ ^39^ ^]^ Copyright 2011, Springer Nature. F) Proteome detection based on the valve chip with cleavage units, enabling simultaneous detection of membrane and secreted proteins. Reproduced with permission.^[^ ^136^ ^]^ Copyright 2021, Mdpi. G) Single‐cell microchamber chip for the detection of up to 42 secreted proteins using multicolor fluorescence and multiplexing strategies. Reproduced with permission.^[^ ^33^ ^]^ Copyright 2015, National Academy of Sciences. H) Secondary isolation based on micromanipulation for monoclonal drug screening. Reproduced with permission.^[^ ^144^ ^]^ Copyright 2011, Springer Nature. I) Secondary isolation based on valve structure for monoclonal drug screening. Reproduced with permission.^[^ ^145^ ^]^ Copyright 2020, Royal Society of Chemistry. J) Secondary isolation based on film punching for monoclonal drug screening. Reproduced with permission.^[^ ^146^ ^]^ Copyright 2019, Royal Society of Chemistry.

The microwell chip captures single cells in microwells, in which the proteins secreted by the cells are captured on glass slides pre‐coated with antibodies and detected by multicolor channels that can detect up to four secreted proteins (**Figure** **8**A).^[^ ^126^ ^]^ This method improves the detection sensitivity and throughput to a certain extent; however, further separation and extraction of target cells requires the use of micromanipulation and other methods.^[^ ^127^ ^]^ Therefore, some studies have used self‐screening microwell array chips to separate cells using hydrodynamics, which improves the throughput and efficiency of cell separation. The target cells of interest were separated by punching. Importantly, because this method immobilizes the antibody on the detection membrane, changing the membrane does not affect the cells in the well and dynamic analysis of secreted proteins can be easily performed (Figure 8B).^[^ ^128^ ^]^ However, the small space of the microwells affects the normal proliferative activity of cells during the detection process, and the single‐detection throughput is also affected by spectral overlap. Some studies have used quenchable magnetic bead encoding, and the detection flux can reach 6 or even higher to overcome spectral overlap,^[^ ^129^ ^]^ whereas others have used mass spectrometry (MS) instead of fluorescence, which greatly improves the detection throughput, reaching thousands.^[^ ^130^ ^]^ Some researchers have expanded the microwell MS detection method to the simultaneous analysis of the proteome and metabolome.^[^ ^131^ ^]^ However, their excessive operation sacrifices cell throughput and detection time. Although MS is currently the gold standard for large‐scale protein characterization, it has limitations in terms of sensitivity and coverage range when dealing with low‐input protein samples, especially in single‐cell proteomics. Compared with MS, nanopore protein sequencing technology offers higher sensitivity and better characterization of protein forms.^[^ ^132^ ^]^ In the future, it is expected that the potential of nanopore protein sequencing technology in single‐cell proteomics will be developed and integrated with micropore array chip technology.

Agarose droplets of functionalized antibody magnetic beads were used to separate and detect the secretion of the three cytokines from single cells. The main limitation is background fluorescence, and there are also studies using droplet microfluidics coated with functionalized antibody magnetic beads to separate and analyze single‐cell proteomics, allowing the simultaneous determination of membrane and secreted proteins(Figure 8C).^[^ ^64^ ^]^ However, both microwells and the aforementioned methods are limited by spectral overlap. Therefore, DNA‐antibody magnetic beads have been used instead of antibody magnetic beads to determine proteomes by sequencing (Figure 8D),^[^ ^133^ ^]^ single‐cell MS‐based shotgun proteomics technology in droplet chips can overcome spectral overlap by replacing antibody fluorescence detection technology.^[^ ^134^ ^]^ But as mentioned above, the droplet environment may affect cell activity and is not suitable for long‐term detection of secreted proteins.

A single‐cell magnetic bead microfluidic valve chip enables the detection of intracellular proteins,^[^ ^135^ ^]^ a single‐cell barcode detection chip based on valve separation (SCBC) can detect multiple secreted proteins at the single‐cell level. This technique demonstrates, for the first time, the multiplexing capabilities of barcode antibodies, enabling quantitative measurement of the secretion of more than ten proteins from a single cell, revealing a high degree of functional heterogeneity even among phenotypically similar T cell populations (Figure 8E).^[^ ^39^ ^]^ To further detect intracellular proteins, some studies have integrated a cell lysis unit (Figure 8F),^[^ ^136^ ^]^ and MS can increase the detection throughput by thousands.^[^ ^125^ ^]^ However, it can only detect intracellular and membrane proteins, and cannot detect secreted proteins. In addition, the valve structure complexity limits the throughput of the cells. To optimize and simplify SCBC, a high‐throughput microchamber array chip that combines fluorescent pathways with spatial barcoding, a multispectral multiplexing strategy, realizes the multiplex detection of 42 immune effector proteins (Figure 8G).^[^ ^33^ ^]^ Compared with the valve‐based method, the complexity of the workflow and the volume of the equipment are reduced, cell viability is better, and cell‐secreted proteins can be dynamically analyzed. However, the problems of low separation efficiency mentioned above can occur, and it is not easy to extract the target cells.^[^ ^35^ ^]^ Single‐cell microchamber chips have been widely used in many fields, such as cancer research,^[^ ^46^ ^]^ cell classification,^[^ ^37^ ^]^ stem cell differentiation,^[^ ^137^ ^]^ and immunology,^[^ ^33^, ^35^, ^138^, ^139^ ^]^ and have also been used to carry out many dual simultaneous analyses of biological information, such as proteins and exosomes^[^ ^46^, ^138^ ^]^ and proteins and RNA.^[^ ^139^ ^]^

Another important application in proteomics, monoclonal drug screening, is very important for the production of highly specific therapeutic antibody drugs, improving the efficacy of disease treatment, and reducing the side effects of drug use. The process first requires secondary screening and separation of isolated single cells to obtain the monoclonal cells of interest, followed by long‐term culture to generate a monoclonal cell population to produce drugs, such as monoclonal antibodies.^[^ ^140^ ^]^ However, the isolation and screening of monoclonal cells remain challenging steps in monoclonal drug screening. Limiting dilution is a routine method for isolating monoclonal cells to produce monoclonal antibodies. As mentioned above, the separation throughput is limited by the well plate; the separation efficiency is limited by the Poisson distribution, which is labor‐intensive and time‐consuming; and the isolation throughput and efficiency limit the development of monoclonal drug screening.^[^ ^141^ ^]^ FACS is a high‐throughput alternative method in which cells are encapsulated in magnetic beads or gel droplets containing capture antibodies, after which the secreted products are detected using fluorescently labeled antibodies, and target cells are screened based on their production levels by flow cytometry.^[^ ^142^, ^143^ ^]^ However, as mentioned previously, picoliter‐level enclosed small spaces pose challenges for medium replacement. The narrow space makes spectral overlap inevitable, and the shear force after separation affects cell activity. These spatial limitations and cell activity issues have hindered the development of this method for monoclonal drug screening. Although label‐free detection methods, such as electrical detection, can solve the problem of spectral overlap, current electrical methods are not yet able to simultaneously detect multiple secreted proteins with high specificity at the same time. In the future, further developments and innovations in electrical detection techniques, such as integrating specific capture probes or developing new sensor materials and structures, are expected to overcome this limitation and achieve high specificity and sensitivity for the detection of multiple secreted proteins.

With the continuous development of the single‐cell isolation chip technology, an increasing number of chips have been developed for monoclonal drug screening. Since the detection of monoclonal drugs generally does not require multiple detections or large amounts of cell space before secondary screening, high‐throughput and efficient single‐cell isolation is required to screen a large number of monoclonal cells. Therefore, low‐quality separation methods are typically used in primary separation to ensure normal ability and a good environment for cell proliferation during secondary screening.

Therefore, some studies have used a microwell array chip designed according to cell size. Compared to the limiting dilution method, the separation throughput and efficiency are greatly improved, and the small space greatly shortens the time for accumulation to the limit of detection. However, the small space does not allow the normal proliferation of monoclonal cells, and it is necessary to use a micromanipulator to retrieve target cells for transfer and further culture, which reduces the efficiency of secondary screening (Figure 8H).^[^ ^144^ ^]^ A microfluidic chip combined with a microwell and valve structure integrates single‐cell separation, detection, transfer, and proliferation on a single chip, thereby saving time and cost. Compared to the traditional limiting dilution method, the total time of 14 days was reduced, and mass proliferation of monoclonal cells was achieved in 5 days (Figure 8I).^[^ ^145^ ^]^ Importantly, the integrated device reduces the possibility of hybridoma mixing and cross‐contamination, ensuring accurate monoclonality; however, throughput is limited owing to the complex valve structure. These methods are primarily used to prepare the cell suspensions. For adherent cells, such as CHO cells, additional enzymatic digestion is required, which affects the overall screening process and results. Another self‐screening chip could be used to produce various types of monoclonal cells (Figure 8J).^[^ ^146^ ^]^ Compared with a microwell chip that separates cells according to size, because the separation of cells relies on hydrodynamic force rather than gravity, it can ensure good single‐cell separation throughput and efficiency, even with pores slightly larger than the cells, and slightly larger pores can also ensure cell activity during the detection process. The dynamic secretion of monoclonal cells can be measured by replacing the detection film, and the target cells can be punched into high‐throughput well plates to screen for clonal proliferation of target cells.

We summarized and divided the current research areas for single‐cell isolation based on microwells, microchambers, droplets, and valve chips in single‐cell proteome analysis, as shown in **Table** **6**. Proteomics directly reflects the phenotype and function of cells and is one of the most effective methods for identifying molecular markers and drug targets for diseases. This requires high cell activity and high detection throughput, especially for secreted protein analysis, which requires long‐term real‐time detection and often requires sufficient space for cell proliferation and detection. Therefore, proteomic analysis typically uses single‐cell separation chip technology with a large space, such as valves or microchamber chips. Considering the separation throughput and efficiency, the microchamber separation chip technology is the best choice. In addition, for monoclonal drug screening, which is an important application of proteomics, two types of separation chips are often sequentially used. The first requires high‐efficiency separation and the second requires high‐activity separation, which is performed immediately. Therefore, valve microchamber or microwell transfer methods are often used.

**Table 6:** Use of single‐cell isolation chip technology for proteome analysis in different fields.

Research field	Chip structure	Detection object	Detection information	Year	Reference
Cancer research	Microwell	HeLa cells	Proteome and Metabolome	2021	[131]
Cancer research	Valve	PC‐9 human lung adenocarcinoma cells	Proteome	2022	[125]
Cancer research	Microwell	Carcinoma NCI‐H1650 cells or CCRF‐CEM leukocytes	Proteome	2017	[127]
Cancer research	Valve	Prostate cancer (LNCaP and PC‐3) cells	Proteome	2022	[135]
Cancer research	Microchamber	Ovarian tumor cells	Proteome and exosomes	2022	[46]
Cell classification	Microchamber	MDA‐MB‐231, K562, 293T, HEY, and SH‐SY5Y cells	Proteome	2022	[37]
Drug treatment	Microwell	Prostate cancer (PCa) cells	Proteome	2021	[128]
Drug treatment	Valve	H1650 lung cancer cells	Proteome	2021	[136]
Drug screening	Microwell	Antigen‐specific antibody‐secreting cells	Proteome	2011	[144]
Drug screening	Valve	Hybridomas	Proteome	2020	[145]
Drug screening	Microwell	VU1D9 hybridoma and CHO cells	Proteome	2019	[146]
Embryology	Droplet	Mouse oocytes	Proteome	2018	[134]
Biomarker discovery	Microwell	Murine cells	Proteome	2021	[130]
Stem cell differentiation	Microchamber	hADSCs	Proteome	2019	[137]
Immunology	Microwell	Primary human T cells	Proteome	2011	[126]
Immunology	Droplet	T and B cells	Proteome	2020	[64]
Immunology	Droplet	Jurkat (clone E6‐1) and Raji cells	Proteome	2017	[133]
Immunology	Valve	Cytotoxic T lymphocytes (CTLs)	Proteome	2011	[39]
Immunology	Microchamber	Human macrophages	Proteome	2015	[33]
Immunology	Microchamber	Human macrophages	Proteome	2019	[35]
Immunology	Microchamber	CAR‐T and lymphoma cells	Proteome	2023	[47]
Immunology	Microchamber	Tumor cells, CAFs, and macrophages	Proteome and exosomes	2023	[138]
Immunology	Microchamber	Mouse macrophages	Proteome and RNA	2016	[139]
Immunology	Microwell	THP‐1 (ATCC TIB‐202) human monocytes	Proteome	2019	[129]

### 3.5 Application of the Single‐Cell Isolation Chip for Single‐Cell Exosome Analysis

Exosomes carry various biomolecules, including proteins,^[^ ^147^ ^]^ RNA,^[^ ^148^, ^149^ ^]^ and DNA,^[^ ^150^, ^151^ ^]^ from parent cells, and play important roles in cell‐to‐cell communication. Studies have shown that exosomes are involved in the pathological processes of various diseases, including cancer, and that their function in cancer development is dynamic.^[^ ^152^ ^]^. In‐depth analysis of exosomes can not only help us understand the function of parent cells, but also achieve early diagnosis of cancer.^[^ ^153^, ^154^ ^]^ There is heterogeneity among cells and secreted exosomes are no exception. To reduce the masking of useful information by population analysis, it is necessary to analyze exosomes at the single‐cell level or even perform an in‐depth analysis of the heterogeneity of exosomes.^[^ ^40^, ^155^ ^]^ Exosomes are similar to secreted proteins and their normal secretion requires high‐quality single‐cell isolation. With the development of microfluidic chip technology, many chip technologies have emerged for controlling and manipulating single cells at the microscale, reducing sample consumption and improving throughput and sensitivity, thereby providing a reliable method for single‐cell exosome analysis.

**Figure 9:**
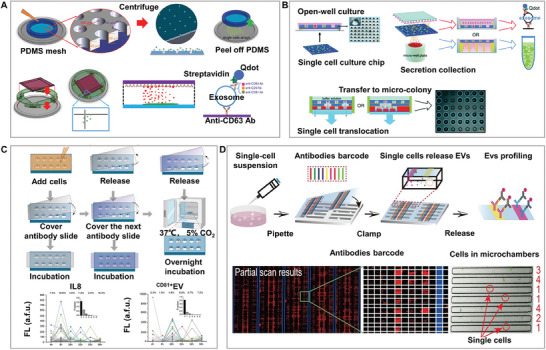
Single‐cell isolation chip for exosome analysis. A) Single‐cell exosome analysis based on peelable microwell structures for quantitative monitoring of individual cell behavior. Reproduced with permission.^[^ ^157^ ^]^ Copyright 2016, Wiley. B) Single‐cell exosome analysis based on microwell structure with mesh for large‐scale parallel single‐cell transference according to user‐defined criteria. Reproduced with permission.^[^ ^158^ ^]^ Copyright 2018, Royal Society of Chemistry. C) Concave microwell structure for the dynamic analysis of single‐cell exosomes, identifying “super secretors” within cell populations. Reproduced with permission.^[^ ^80^ ^]^ Copyright 2021, Cold Spring Harbor Laboratory. D) Single‐cell exosome analysis based on the microchamber chip, marking the first multiplex barcode analysis of extracellular vesicle (EV) secretion from single cells, offering new insights on intercellular communication. Reproduced with permission.^[^ ^161^ ^]^ Copyright 2020, American Chemical Society.

Microwell chips are the most commonly used single‐cell exosome detection platforms; as mentioned above, they exhibit insufficient activity in isolated cells. Some studies have reported a unique detachable microwell microfluidic device that can be mechanically peeled after single‐cell isolation. Single cells continue to grow in normal culture dishes, and a sufficient supply of culture medium and growth space ensures their activity of single cells. This platform enables the continuous monitoring of extracellular matrix interactions and exosome secretion in single cells, providing important quantitative data for an in‐depth understanding of cell behavior. However, because the cells are in a non‐enclosed space, although the method has been reported to cause a small amount of crosstalk (3%), the detection multiplicity is still limited by the detection principle (**Figure** **9**A).^[^ ^156^ ^]^ The multiplicity of direct on‐chip exosome detection is also affected by spectral overlap, which does not permit a comprehensive dissection of the heterogeneity of exosome secretion. Therefore, microwell chip technology with a micromembrane beneath it was developed. Microwell chips can be sized to filter exosomes and perform downstream enrichment detection, and PDMS can be patterned using UV light to transfer the target single cells to another single‐cell culture chip (Figure 9B).^[^ ^157^ ^]^ In addition, there some studies have extracted exosomes from target micropores for detection.^[^ ^158^ ^]^ Subsequent studies on microwell chips have detected exosomes using label‐free methods, such as electrical detection, to overcome the effects of spectral overlap. However, as mentioned earlier, microwell chips may affect the normal activity of cells because of the limited space.

Droplet chips are commonly used for single exosome detection. This article mainly discusses the isolation and application of single cells, and further single‐vesicle isolation and applications are not discussed in detail here.^[^ ^159^ ^]^ In addition, to ensure the normal activity of cells, some studies have been performed to appropriately increase the size of microwells and design small‐port packaging, which can reduce the loss of cells when the detection chip is replaced for the continuous measurement of dynamic changes. This microchip was used for continuous multi‐day tracking of interleukin‐8 and CD81^+^ extracellular vesicle (EV) secretion from the same single cell, revealing the presence of supersecretors within the cell populations (Figure 9C).^[^ ^80^ ^]^ The efficiency of cell isolation was reduced, but the large space ensured normal cell activity. High‐activity microchamber array chips can overcome these limitations and may be the most suitable platform for exosome detection at present (Figure 9D).^[^ ^161^ ^]^ The space is large enough, and the multiplexed barcode array overcomes spectral overlap. Using a spatially distributed antibody barcode microfluidic platform, multiplex analysis of EV secretion from over 1000 single cells was achieved for the first time, revealing unprecedented single‐cell heterogeneity in EV secretion among human oral squamous cell carcinoma cells. This is an indispensable tool for single‐cell analysis and EV research. Subsequently, this method has been applied in immunology.^[^ ^138^ ^]^ and cancer research.^[^ ^46^ ^]^ This method can simultaneously detect exosomes and secreted proteins in single cells; however, because of the closed environment, subsequent enrichment detection cannot be performed. Additionally, when performing long‐term or continuous detection, it is necessary to remove the detection substrate and replace it with a new substrate, which may cause cell loss.

We summarized and divided the current research areas for single‐cell isolation based on microwells and microchamber chips in single‐cell exosome analysis, as shown in **Table** **7**. In general, exosomes carry various biomolecules from parental cells and play an important role in intercellular communication. Their normal secretion requires normal cell activity; therefore, their application usually uses separation methods with good cell activity, such as single‐cell microchamber isolation or large‐sized microwell isolation. For applications with high detection throughput requirements, single‐cell microchamber chips can be used to avoid the influence of spectral overlap. Large micropores can be used for devices with low detection throughput requirements.

**Table 7:** Use of single‐cell isolation chip technology for exosome analysis in different fields.

Research field	Chip structure	Detection object	Detection information	Year	Reference
Cancer research	Microwell	MCF‐7 and MCF‐10A cells	Exosomes	2023	[158]
Cancer research	Microchamber	Ovarian tumor cells	Proteome and exosomes	2022	[46]
Cancer research	Microchamber	OSCC cells	Proteome and exosomes	2019	[40]
Drug screening	Microwell	MCF10A, MCF7, and MDA‐MB‐231 cells	Exosomes	2016	[156]
Microbiology and biomarker discovery	Microwell	Glioblastoma and microcolonies	Exosomes	2018	[157]
Immunology	Microwell	SCC25 and human monocytic U937 cells	Exosomes	2022	[80]
Immunology	Microchamber	Tumor cells, CAFs, and macrophages	Proteome and exosomes	2023	[138]

The current challenges in single‐cell exosome analysis are mainly due to three aspects listed below. (1) Capture efficiency: When capturing exosomes on a chip, the common transmembrane protein antibody CD63 is often used, but the exosomes secreted by single cells are in a free state on the chip, presenting a problem of contact probability. Dielectrophoresis and photothermal enrichment of exosomes onto the capture substrate are potential methods to improve the capture efficiency of single‐cell exosomes suitable for the directional enrichment of exosomes, which are small particles with biomembranes, thereby enhancing the detection efficiency. (2) Detection sensitivity: Compared to traditional methods, such as ultracentrifugation for enriching cell groups or serum exosomes, the amount of exosomes secreted by single cells is small, requiring extremely high detection sensitivity. Although microfluidic chips have improved the detection sensitivity compared to traditional methods, exosome detection still poses challenges, necessitating more sensitive substrates and detection methods. Methods for improving the exosome detection sensitivity include reducing the reaction space (e.g., using micropore chips or droplet chips) and using highly sensitive sensing substrates (e.g., GOQD‐based microcavity chips) combined with label‐free detection methods (e.g., electrical) to overcome the impact of spectral overlap. (3) Detection limitations: Currently, the detection of single‐cell exosomes based on microfluidic chips is limited to exosome phenotypes, such as exosome size and membrane proteins. In future, microfluidic technologies capable of detecting antigens, DNA, or RNA within single‐cell exosomes should be developed for a deeper understanding of intercellular communication and disease mechanisms.

## 4 Discussions and Prospects

### 4.1 Achievements and Weaknesses

#### 4.1.1 Single‐Cell Chip Multiomic and Joint Exosome Analysis

Single‐cell isolation chip technologies and applications demonstrate chip‐based capabilities for single‐cell analysis and provide new avenues for understanding individual cells. Although these technologies have made significant progress, chip‐based integration and analysis of diverse single‐cell biological information still faces some challenges. The integration of single‐cell omics data can help us gain a more comprehensive and accurate understanding of the complexity and diversity of biological systems, enabling deeper insights into cellular functions and regulatory mechanisms. It can help explain cell heterogeneity, illuminate cell development and differentiation, identify novel biomarkers, explore transcriptional regulatory mechanisms, discover new biological modules and pathways, and investigate complex diseases, among other applications, offering enormous potential for future development. Currently available technologies for combining various biological information include genome and transcriptome sequencing.^[^ ^160^ ^]^ ATAC‐seq^[^ ^161^, ^162^ ^]^ and single‐nucleus chromatin accessibility and mRNA expression sequencing.^[^ ^163^ ^]^ for integrating genomic, epigenomic, and transcriptomic data, as well as cellular indexing of transcriptomes and epitopes by sequencing.^[^ ^164^, ^165^ ^]^ RNA expression and protein sequencing assay,^[^ ^166^ ^]^ paired‐tag,^[^ ^167^, ^168^ ^]^ and surface‐protein glycan and RNA‐seq.^[^ ^169^ ^]^ for combining transcriptomic and proteomic data. CODEX enables the integration of proteomic data and cell phenotypes,^[^ ^170^ ^]^ while the scTrio‐seq technique combines three types of biological information, simultaneously measuring the genomic DNA, transcriptomic RNA, and protein expression of a single cell.^[^ ^171^ ^]^ These integrated techniques can help to identify genomic variations, gene expression disparities, and protein expression levels within individual cells, leading to a deeper understanding of cellular diversity and complexity.

Currently, single‐cell EV analysis in combination with multiomics is an emerging area of research aimed at integrating and correlating individual cell EV information with other biological information, such as genomics, epigenomics, transcriptomics, and proteomics. Considering the crucial role of EVs in cell communication and regulation, combining EVs with other omics data can provide insights into intercellular interactions and signal transmission. Although single‐cell multiomics and EV joint analysis present a complex task, they are continuously evolving with emerging potential techniques and approaches, such as scATAC‐seq and exosome joint analysis,^[^ ^172^, ^173^ ^]^ scRNA‐seq and exosome joint analysis,^[^ ^172^ ^]^ and secreted protein and EV phenotypic joint analysis.^[^ ^40^, ^46^ ^]^ However, the technology for single‐cell EV and multiomics joint analysis is still under development and faces certain technical challenges, such as EV capture and isolation, data processing, and integration. With continued technological advancements and methodological developments, more powerful tools and strategies are expected to emerge, providing deeper insights into the relationship between EVs and cellular functionality.

#### 4.1.2 Selection of Single‐Cell Isolation Technologies for Multiomic and Exosome Analysis

In‐depth analysis of vast amounts of data is essential for unraveling complex cell characteristics and functions in single‐cell multiomics and EV research, making high‐throughput and efficient single‐cell separation critical. However, simultaneously achieving high throughput, efficiency, cell viability, and spatial requirements during single‐cell isolation remains challenging. Hence, distinct separation technologies are preferred to meet the specific demands of different applications, such as single‐cell genomics, epigenomics, transcriptomics, proteomics, and EV analysis. Single‐cell microwells and droplet chips with high throughput and efficiency are commonly employed for single‐cell genomics, epigenomics, and transcriptomics, where immediate cell fixation usually requires post‐separation and cell activity is less crucial. The smaller space in these platforms not only reduces reagent consumption, but also enhances reaction efficiency. These technologies have been proven effective for single‐cell genome, epigenome, and transcriptome applications.^[^ ^83^, ^86^, ^95^, ^100^, ^109^, ^116^ ^]^ Nanopore sequencing technology is a label‐free electrical detection method that does not require PCR amplification. Its unique advantages hold immense potential for applications in single‐cell genomics,^[^ ^85^ ^]^ epigenetics,^[^ ^106^, ^107^ ^]^ and transcriptomics.^[^ ^111^, ^117^ ^]^ In the future, microfluidic technology is expected to be further integrated, opening new possibilities for single‐cell multiomics analyses. However, proteomics and exosome analysis require high cell viability and space to ensure the activity of isolated cells during culture. In such cases, single‐cell microchambers and valve chips with large spaces are preferred.^[^ ^33^, ^39^, ^40^, ^136^, ^145^ ^]^ These platforms allow long‐term culture with ample nutrition, facilitate spatial multiplexing, minimize spectral overlap, and increase detection throughput. Although transferring cells to a larger space after secondary separation after micropore separation is a viable option, it has certain limitations, such as potential spectral overlap, challenges in balancing flux and efficiency, limited application expandability, and the risk of secondary pollution. In conclusion, the choice of separation technology for single‐cell analysis should be tailored to the specific requirements of its application. Single‐cell microwells and droplet chips are suitable for high‐throughput single‐cell genomics, epigenomics, and transcriptomics, whereas single‐cell microchambers and valve chips are more appropriate for single‐cell proteomics and exosome analysis, considering the need for higher cell viability and spatial requirements.

#### 4.1.3 Preservation of Spatial Information

During single‐cell isolation, researchers must be attentive to preserving both the spatial information and the original location of the cells. In most single‐cell isolation chip studies, cell dissociation from the tissue precedes single‐cell isolation, resulting in the loss of the first layer of spatial information within the tissue. Although spatial omics can retain this spatial information, a single‐cell resolution has not yet been achieved.^[^ ^174^, ^175^, ^176^, ^177^ ^]^ Additionally, there are certain challenges associated with single‐cell throughput. Some single‐cell isolation chip technologies are incapable of conducting subsequent in situ analyses, leading to a loss of in situ cell information after isolation. This issue is of paramount importance for applications that require in situ information, such as drug screening, cell‒cell interactions, and immune evaluations. Hence, the choice of single‐cell isolation technology must be based on the specific demands of the downstream applications. Microvalves, microchambers, and microwell chips are usually capable of preserving single‐cell in situ information, whereas droplet microfluidic separation techniques may lose such information.

#### 4.1.4 Improvement in Cell Activity

Cell activity is characterized by dynamic changes, and preserving the normal physiological functions of cells is crucial, particularly in the analysis of secreted proteins and exosomes. Although various separation and analysis applications provide diverse information on single cells, most tissue cells are in an adherent state before dissociation and in a suspended state during analysis, potentially leading to a loss of essential morphological and proliferative characteristics. Hence, new single‐cell separation chip technologies that ensure the maintenance of normal morphology and proliferation of adherent cells need to be developed. Current approaches involve the use of bioaffinity on the surface of microcavity chips to promote normal morphology and proliferation of adherent cells.^[^ ^37^, ^178^, ^179^ ^]^ and the implementation of a replaceable fluid system to preserve the physiological activity of cells.^[^ ^180^ ^]^ This structure is advantageous for in situ cultures and can be used to study the mechanism of drug addition to the culture medium to stimulate single cells.^[^ ^43^, ^44^, ^45^, ^181^ ^]^ However, these studies were ultimately constrained by the challenge of insufficient cell separation efficiency owing to the Poisson distribution. An alternative approach is the use of droplet chips that overcome the Poisson distribution by enabling adherent cells to grow on hydrogel balls within the droplet.^[^ ^182^ ^]^ This unique structure facilitates supplementation of the culture medium; however, fluorescence detection may suffer from spectral overlap. Another technique involves the secondary separation of high‐throughput microwell chips into well plates or microreaction chambers to support the normal growth and proliferation of adherent cells.^[^ ^81^ ^]^ However, this method raises concerns regarding potential secondary pollution. In the future, an optimal solution might involve combining these separation methods to enable effective proteomic and exosome analyses of adherent cells, thereby mitigating the challenges associated with individual techniques. Careful integration of these approaches may offer a promising pathway for maintaining normal cell activity during single‐cell separation in advanced proteomic and exosome studies.

### 4.2 Future Prospects

Currently, microfluidic chips are being rapidly developed. Moreover, different types of single‐cell separation chip technologies are available for different single‐cell information analyses, as summarized in this article. In the future, a new technology integrating two or more single‐cell separation chip technologies may be developed and used for joint single‐cell multiomic and exosome analyses. However, many limitations need to be overcome, such as the contradiction between the throughput efficiency and spatial activity of the separation chip, which needs to retain the spatial information of the cells on the chip to trace the target cells, and good cell viability in joint analysis, which needs to be ensured for accurate proteomic and exosome information. Such new multi‐information single‐cell analysis technologies can further promote the rapid development of various fields, including cancer research, drug development and screening, immunology, biomarker discovery, cell classification, and microbiology, and facilitate the exploration of the functional mechanisms of single cells, making significant contributions to public health.

## Conflict of Interest

The authors declare no conflict of interest.

## Author Contributions

C.W. wrote the manuscript draft. J.Q. and M.L. drew pictures and inserted the references. Y.W., Y.Y., H.L., Y.Z., and L.H. reviewed and edited the manuscript.
